# Are all patches worth exploring? Foraging desert birds do not rely on environmental indicators of seed abundance at small scales

**DOI:** 10.1186/s12898-019-0242-z

**Published:** 2019-06-18

**Authors:** Fernando A. Milesi, Javier Lopez de Casenave, Víctor R. Cueto

**Affiliations:** 1 Desert Community Ecology Research Team (Ecodes), Departamento de Ecología, Genética y Evolución, Facultad de Ciencias Exactas y Naturales, Universidad de Buenos Aires, and IEGEBA (UBA–CONICET), Piso 4, Pabellón 2, Ciudad Universitaria, Buenos Aires, Argentina; 2 Present Address: Grupo de Ecología Terrestre de Neuquén (CONICET–CEAN), Instituto de Investigaciones en Biodiversidad y Medio Ambiente, INIBIOMA (CONICET–UNCo), Junín de los Andes, Neuquén Argentina; 3 Present Address: Centro de Investigación Esquel de Montaña y Estepa Patagónica, CIEMEP (UNPSJB–CONICET), Esquel, Chubut Argentina

**Keywords:** Post-dispersal seed predation, Field experiment, Granivory, Patch exploration, Patch exploitation, Soil seed bank, Vegetation structure, Environmental cues, Predation risk, Arid environments

## Abstract

**Background:**

Consumers should show strong spatial preferences when foraging in environments where food availability is highly heterogeneous and predictable. Postdispersal granivores face this scenario in most arid areas, where soil seed bank abundance and composition associates persistently with vegetation structure at small scales (decimetres to metres). Those environmental features should be exploited as useful pre-harvest information, at least to avoid patches predicted to be poor. However, we did not find the expected spatial association in the algarrobal of the central Monte desert by observing foraging seed-eating birds, a field technique influenced by how much they exploit visited patches. In this work we tested if the first stage of foraging by granivorous birds (patch visit, encounter or exploration) is positively associated with environmental indicators of patch quality by recording the removal of single seeds from 300 scattered experimental devices during seasonal trials. Spatial selectivity was analysed by comparing the structural characteristics of used vs. available microhabitats, and evaluated against bottom-up and top-down hypotheses based on our previous knowledge on local seed bank abundance, composition and dynamics. Their foraging activity was also explored for spatial autocorrelation and environmental correlates at bigger scales.

**Results:**

Postdispersal granivorous birds were less selective in their use of foraging space than expected if microhabitat appearance were providing them relevant information to guide their search for profitable foraging patches. No microhabitat type, as defined by their vegetation structure and soil cover, remained safe from bird exploration. Analyses at bigger temporal and spatial scales proved more important to describe heterogeneity in seed removal.

**Conclusions:**

Closeness to tall trees, probably related to bird territoriality and reproduction or to their perception of predation risk, seemed to determine a first level of habitat selection, constraining explorable space. Then, microhabitat openness (rather than seed abundance) exerted some positive influence on which patches were more frequently visited among those accessible. Selective patterns by birds at small scales were closer to our predictions of a top-down spatial effect, with seed consumption creating or strengthening (and not responding to) the spatial pattern and dynamics of the seed bank.

**Electronic supplementary material:**

The online version of this article (10.1186/s12898-019-0242-z) contains supplementary material, which is available to authorized users.

## Background

The decision-making process of foraging animals involves gathering information on critical factors, either by inference from reliable environmental cues or by local assessment after a site has been visited [1–3]. Food availability, together with costs and benefits related to foraging efficiency, vulnerability to predators and microclimate are usual candidate factors affecting selection of foraging patches by small individual animals [4–6]. The ability to assess and respond to patchiness at small scales allows foragers to exploit their habitat more efficiently, particularly if physical cues associate with patch boundaries and can be perceived through direct sensory input [7–9]. Consumers should show strong spatial preferences when foraging in environments where food availability is highly heterogeneous and predictable from its correlation with informative environmental features. This would allow them to behave more like prescient foragers, releasing them from the costs of random or indiscriminate exploration (reducing “the penalty of ignorance”) or continuously gathering spatial data to update their knowledge on distribution and location of food patches [2, 10–16].

Seed availability in the soil of most deserts is highly heterogeneous at small scales (e.g., decimetres to meters) and associates persistently with vegetation structure (e.g., [17–20]). The central Monte desert (Argentina) is no exception: seeds and litter consistently accumulate under shrubs and trees [21–24]. In these habitats, visual foragers such as granivorous birds should increase their foraging success by exploiting woody cover and litter, conspicuous environmental cues of resource abundance, as pre-harvest or prior information accumulating their previous experiences and evolutionary history [2, 11, 16].

Foraging processes have been traditionally studied as a sequence of different stages [15, 25]. Postdispersal (from the soil) seed predation can be split in (1) patch visit, encounter or exploration, or which of the available patches are visited to explore for food, frequently stated as the probability of at least one seed being removed, and (2) patch exploitation, the amount or proportion of seeds removed once the patch was visited and before quitting it (“seed encounter” and “seed exploitation” sensu [26]). As predicted by classic “attack” foraging models, like the “optimal patch choice” or “diet” models [27–29], birds should rely on patch appearance, detectable from afar, to attack “good patches” and neglect “poor patches”, saving evaluation costs. However, we did not find this spatial association when we observed granivorous birds foraging on the ground of the central Monte desert: post-dispersal seed consumption (either by bird species or aggregated as a guild) occurred across all the range of available microhabitats, with no detectable differences in vegetation characteristics between used and random patches [30]. A caveat remains: when randomly-searched, the probability of encounter with a foraging organisms at a particular site increases with the time they spend there. In consequence, the derived description of their use of habitat depends not only on the patches they visit but on how long they stay in a visited patch before giving up; i.e., the stage of patch exploitation. In fact, most studies on seed removal ignore or alter the patch searching stage through explicit or implicit baiting (e.g., proper baiting, training sessions, ad libitum patches) to minimize the probability of focal organisms disregarding the experimental setup. In this work we offered scattered, single seeds in different microhabitats to detect where do birds search for them and not how much they are able to remove (or for how much time) when an extraordinary rich patch is suddenly available (the “magic pudding effect”: [31]). Though not frequently, some studies have used, distinguished or compared both approaches (e.g., [25, 32–34]).

Foraging decisions, informed or not by environmental cues, may have ecological consequences [35] if selective consumption influences the spatial distribution of resources, i.e., a top-down spatial effect. Non-random seed removal affects the composition and heterogeneity of the seed bank, so spatial patterns of seed abundance on the ground and, later, of adult plants may be interpreted also as the consequence—instead of the cause—of granivory [36–42]. Most studies on the impact of granivores on desert seed banks have emphasised the impact of rodents and ants (e.g., [17, 43–45]), presumably as an historical consequence of the “irrelevance” of birds on deserts of the Northern Hemisphere ([46–48], but see [32, 38, 49, 50]). However, seed-eating birds should be able to modify abundance, composition and spatio-temporal heterogeneity of the soil seed bank in arid areas where they are important granivores, especially because their diet is usually selective (e.g., [51–54]). In the Monte desert, postdispersal granivorous birds are important consumers of their preferred grass seeds, especially in autumn–winter [53–56]. In that season, granivores may account for up to 50% of grass seed loss in open microhabitats, in coincidence with observed differences between their potential and effective seed banks [55–58]. Though total seed bank is always more abundant under woody vegetation, grass seeds tends to homogenize among microhabitats with time after primary dispersal [22]. Local seed-eating birds, as expected, prefer to exploit more profitable patches once assessed [23, 59], so this top-down effect on the seed bank calls for granivorous birds that do not rely on environmental cues of seed abundance to only attack richer patches but, instead, preferentially explore open, bare-soil microhabitats (at least during autumn–winter).

In this work we evaluated spatial selectivity at small scales by foraging postdispersal granivorous birds in open woodlands (algarrobales) of the central Monte desert. Every season, we analysed the spatial pattern of single-seed removal from 300 experimental devices (arranged every 5 m in three 10 × 10 grids) and compared the structural characteristics of the vegetation in used microhabitats against those available. Our bottom-up hypothesis poses that foragers rely on environmental cues of seed abundance in the soil to only visit high-quality patches. According to our knowledge on local seed-bank dynamics [21, 22, 60], it predicts birds that remove experimental seeds preferentially from devices under shrubs and trees or under grass cover (where seeds concentrate), neglecting those in open patches. On the other side, our top-down hypothesis poses that seed consumption by birds generates or reinforces the spatial heterogeneity at small scales of the seed bank, particularly the observed postdispersal reduction in the density of their preferred seeds in open patches; it predicts granivorous birds that preferentially explore open, bare-soil microhabitats, at least during autumn and winter. Use of space at small scales may depend on, or be constrained by, context at bigger spatial scales, a key knowledge for proper interpretation of selective patterns [61–63]. We have observational evidence on the relevance of tall trees on these foraging birds [30], so seed removal from our experimental devices was explored for spatial patterns and environmental correlates at bigger scales.

## Results

**Fig. 1:**
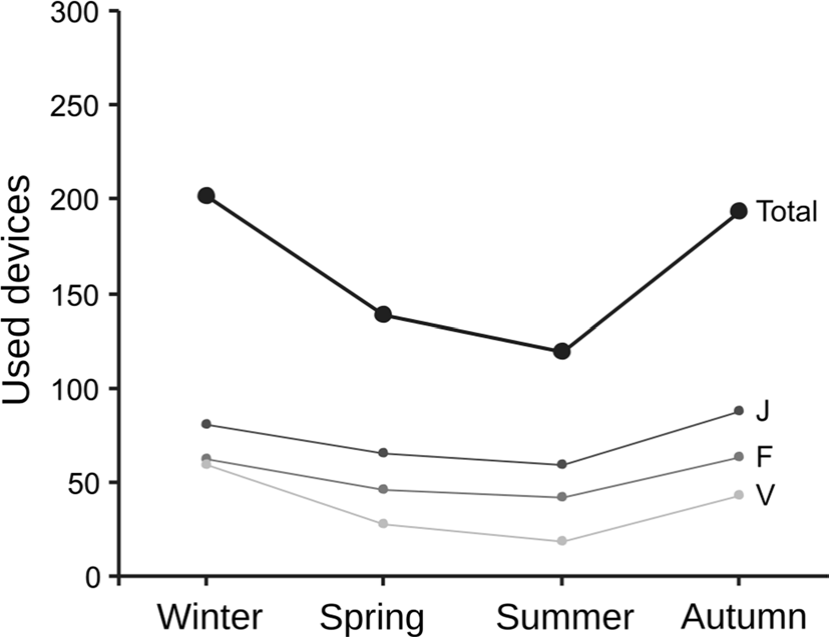
Total seed removal. Number of devices where the seed was removed in at least one of the 2 days offered per season and grid. Total number of devices available was 300 in each season, arranged in three 10 × 10 grids (J, F, V)

The number of devices where the single seed was removed varied among seasons and grids. Seed removal was higher in autumn and winter and decreased in spring to summer in each of the three grids, which showed a consistent relative level of use: grid J had always higher seed removal, followed by grid F, and then grid V (Fig. 1). Both Season and Grid (and not their interaction) were relevant predictors of the number of devices used, with the spatial position at Grid scale an even stronger predictor than Season (Table 1).

**Table 1:** Spatio-temporal heterogeneity of seed removal

Model (~ predictors)	Residual d.f.	Residual deviance	Test	Δ deviance [d.f]	*P*
1. Full model (~ season * grid)					
2. *Main effects model (~ season + grid)*	*6*	*10.423*	*2 vs. 1*	*10.423 [6]*	*0.108*
3. Temporal model (~ season)	8	124.506	3 vs. 2	114.083 [2]	< 0.001
4. Spatial model (~ grid)	9	83.665	4 vs. 2	73.242 [3]	< 0.001
5. Null model	11	191.038	5 vs. 2	180.614 [5]	< 0.001

Analysis of deviance of nested binomial Generalised Linear Models (GLMs; *logit* link) obtained through backwards elimination from the full model, with temporal (Season, four categories) and spatial (Grid, three categories) heterogeneity as predictors of the proportion of used devices in each experiment. The minimum adequate model includes both main effects (model 2, in italics)

Seed removal from a particular device in a grid was not independent among seasonal trials (J: ${\upchi}^{2}{_{4}}$ = 80.23, F: ${\upchi}^{2}{_{4}}$ = 19.90, V: ${\upchi}^{2}{_{4}}$ = 42.02; all: *n* = 100, *P* < 0.001). There were more observations in the categories “always used (4/4)” and “never used (0/4)” than expected if seed-offer devices in a grid were randomly and independently found each season.

### Vegetation and soil characteristics

**Fig. 2:**
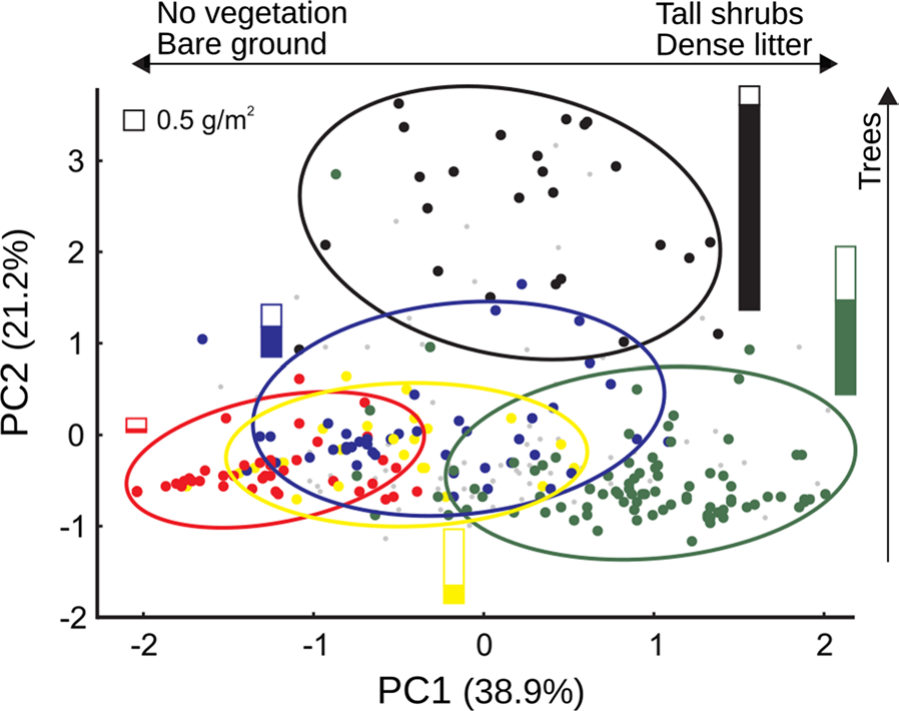
Microhabitat characteristics around seed-offer devices. Position of the 300 seed-offer devices according to the first two components of a PCA on ten vegetation and soil variables measured at the microhabitat scale. Microhabitats are identified by their a priori categorization at field according to previous studies of the soil seed bank [21, 22]: beneath tree canopy (black), beneath tall shrubs (green), beneath low shrubs (blue), beneath grasses (yellow) and bare soil (red). Smaller gray points were microhabitats with intermediate characteristics according to those criteria. Ellipsis include at least 90% cases in each category. Bars show the biomass of forb (full) and grass (open) seeds of each microhabitat category during the same winter of the field experiment (L. Marone, unpublished data). Relative sizes of bars look similar when number of seeds, instead of biomass, was represented (0.5 g/m^2^ ≈ 2000 seeds/m^2^; not shown)

The first three components of a Principal Components Analysis (PCA) on ten characteristics of vegetation and litter measured at the microhabitat scale (around seed-offer devices) retained 73% of the variability in the correlation matrix (Table 2). The first component (PC1) represented general vegetation cover, with positive values associated with tall shrubs and litter and negative values with open areas and bare ground. The second (PC2) was associated with tree cover (to the positive values), and the third (PC3) with the rest of perennial vegetation: grasses to the positive and low shrubs to the negative values. These components can be associated with the soil seed bank according to previous local results based on sampling microhabitats categorized a priori at field: total seed abundance in the soil should be associated with positive values of PC1 and PC2, and grass seed abundance to positive values of PC3 (Table 2, Fig. 2).

**Table 2:** Principal Components Analysis of vegetation and litter characteristics at the microhabitat scale

	PC1	PC2	PC3
Grasses	0.334	*−* 0.138	*0.648*
Dry standing grasses	0.085	0.061	*0.752*
Low shrubs	0.502	0.339	*−* *0.435*
Tall shrubs (< 1 m)	*0.789*	*−* 0.392	0.129
Tall shrubs (> 1 m)	*0.767*	*−* 0.396	0.133
Trees (< 1 m)	0.017	*0.850*	0.040
Trees (> 1 m)	0.121	*0.865*	*−* 0.128
No vegetation	*−* *0.872*	*−* 0.162	*−* 0.176
Bare ground	*−* *0.875*	*−* 0.301	*−* 0.167
Dense litter	*0.877*	0.292	0.094
Eigenvalue	3.892	2.121	1.295
% variability explained	38.9	21.2	12.9
% variability accumulated	38.9	60.1	73.1

Results of the Principal Components Analysis of the ten variables measured at the microhabitat scale. Main loadings for the three retained components are shown in italics

The three grids were generally similar in their vegetation and soil characteristics at this scale though differed slightly on the distribution of their microhabitats over some PCA components: there were more microhabitats covered with low shrubs in V and with grasses in F (low and high values in PC3, respectively), and some more devices under trees in J than in V (PC2) (Kruskal–Wallis tests, *k* = 3, *n* = 300; PC1: *H* = 0.48, *P* = 0.78; PC2: *H* = 5.28, *P* = 0.07; PC3: *H* = 21.68, *P* < 0.01). Mean distances from devices to nearest tall trees showed higher differences among grids: devices in grid J were closer in average to a tree > 3 m high (distance to nearest tree: 2.69 ± 2.27 m), F was intermediate (3.49 ± 3.05 m), and V devices were farther away in average (6.66 ± 4.45 m; K–W test: 48.69, *P* < 0.001). A similar though slightly stronger pattern was found for distances to only the tallest algarrobos (> 4 m high; J: 5.60 ± 4.22 m; F: 8.63 ± 5.51 m; V: 13.18 ± 7.83 m; K–W test: 55.39; *P* < 0.001). Since this pattern matched observed levels of seed removal among grids, selection of characteristics associated with the devices were explored in analyses both pooling grids (i.e., analysis at the habitat extent) and within each grid.

### Foraging site selection based on microhabitat characteristics

**Fig. 3:**
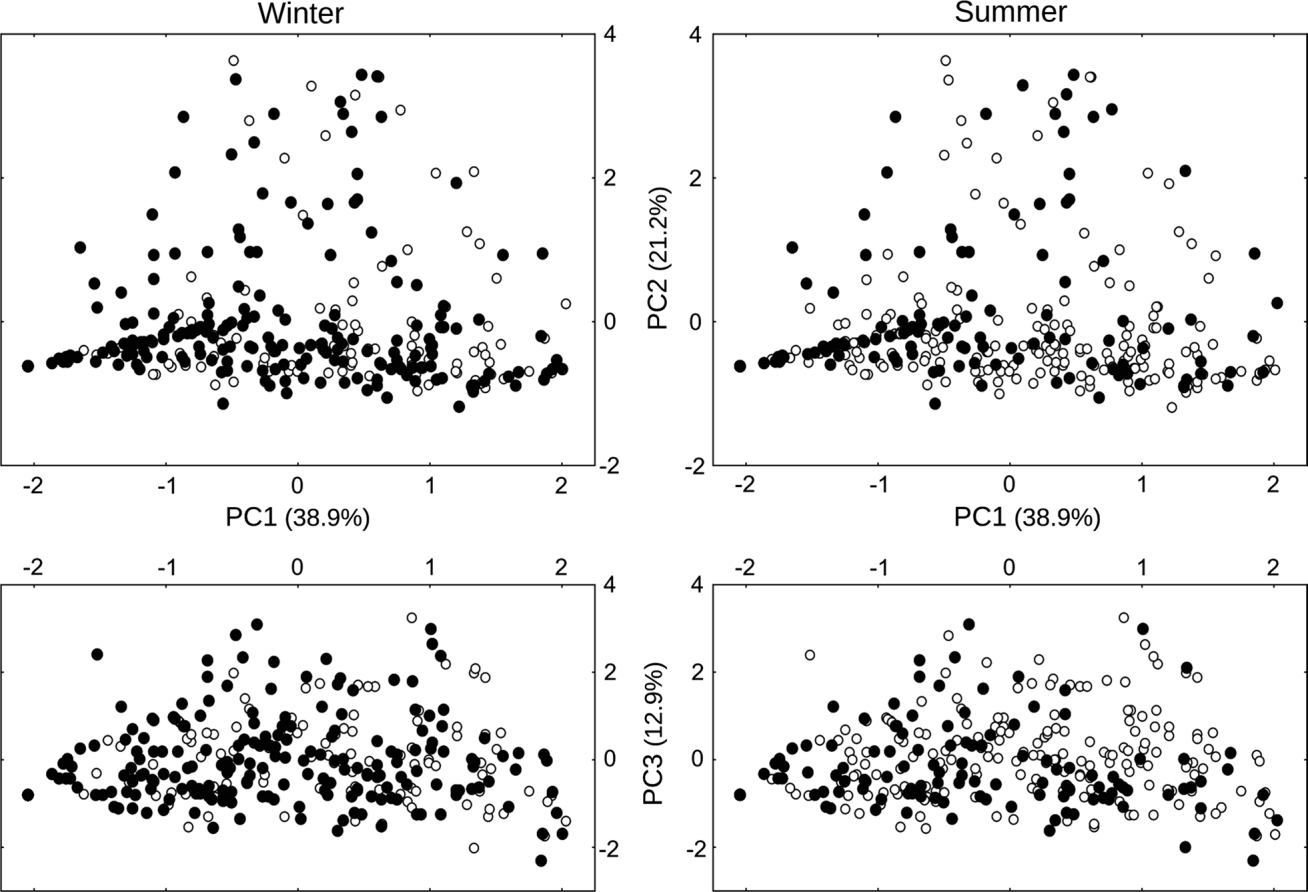
Seed removal according to microhabitat characteristics. Distribution of used (filled circles) and unused (open circles) devices in the multidimensional space of the three first components of a PCA on vegetation and soil characteristics at the microhabitat scale in the two seasonal trials with more (winter) and less (summer) seed removal. Point patterns in autumn were similar to those in winter, and in spring to summer’s (not shown)

There were used devices across all the multivariate space defined by the three PCA components on vegetation and soil characteristics at the microhabitat scale (Fig. 3). However, seed removal was not completely random: used devices in spring and summer (pairs N_11_) and non-used devices in spring (pairs N_00_) were more aggregated than expected by chance (Table 3).

**Table 3:** Segregation of used and non-used devices according to microhabitat characteristics

	Observed/expected	*C* (*Z*)	*P*
Winter		0.271	0.873
N_11_	131/134.5	(− 0.510)	0.610
N_00_	32/32.5	(− 0.083)	0.934
Spring		12.747	0.002
N_11_	*85/63.2*	*(3.380)*	*0.001*
N_00_	*103/87.2*	*(2.328)*	*0.020*
Summer		5.895	0.052
N_11_	*61/47.0*	*(2.339)*	*0.019*
N_00_	119/109.0	(1.464)	0.143
Autumn		2.452	0.293
N_11_	114/123.9	(− 1.457)	0.145
N_00_	38/37.9	(0.012)	0.991

Analysis of segregation of used and non-used devices in the 3D-space generated by the first three components of a PCA on ten variables measured at microhabitat scale. The observed and expected number of pairs of nearest-neighbour points that were both used (N_11_) or non-used (N_00_) in each seasonal experiment are shown, together with the statistic (C) testing for global spatial segregation between classes of points against the null hypothesis of “random labelling”, and the statistic (Z, between brackets) testing the same hypothesis for each kind of pair. A significantly higher number of pairs observed than expected indicates positive spatial correlation (aggregation, in italics)

**Fig. 4:**
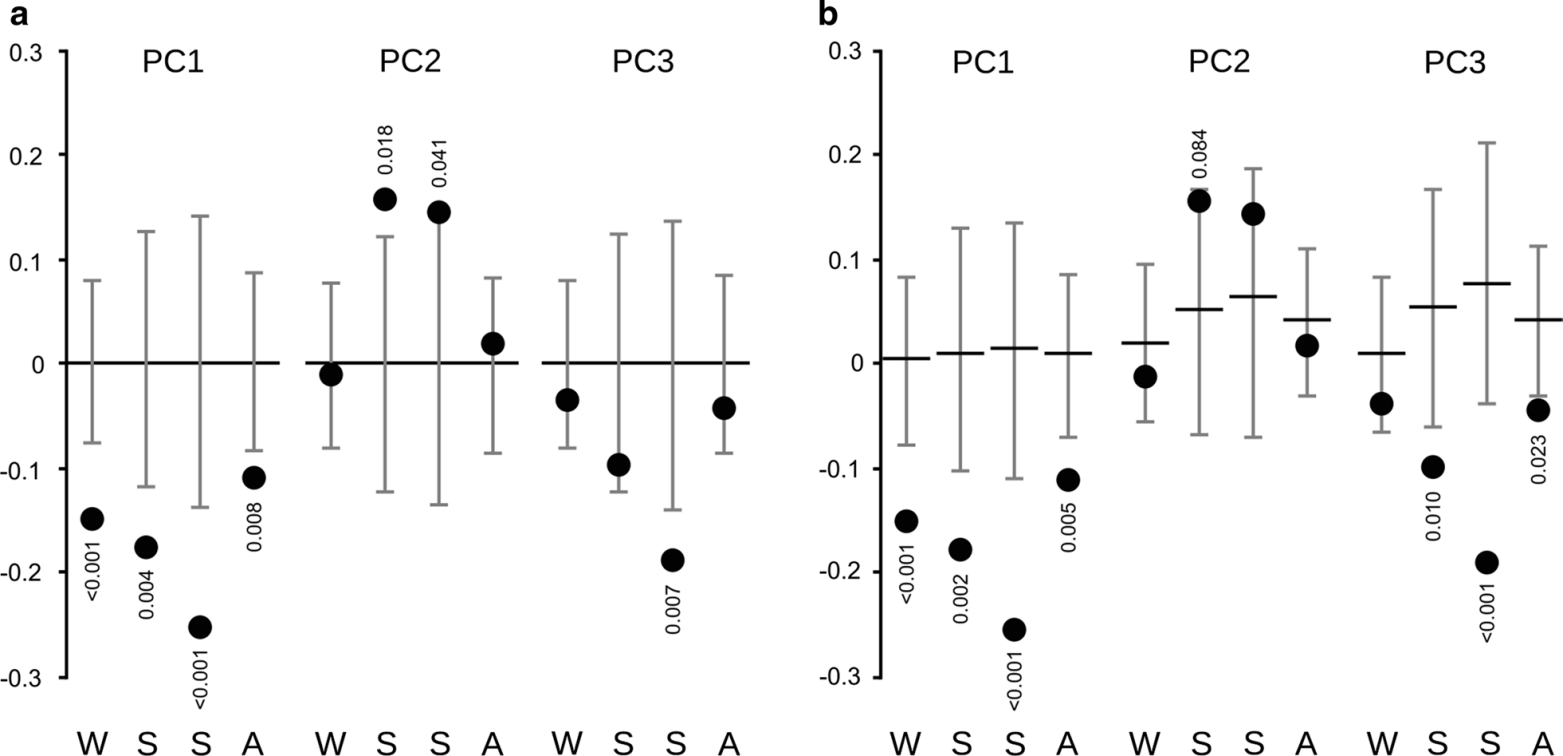
Selection of patch characteristics at the microhabitat scale. Mean scores of used devices in the first three components of the PCA (black circles) in each season (from winter [W] to autumn [A]), controlling (**b**) or not (**a**) for potential selective use of space at the grid scale. Black horizontal line is the expected value and whiskers show the 95% confidence interval under the null hypothesis of no selection. Pseudo P-values not shown when *P* > 0.1 (two-tailed test)

Unidimensional analysis along each PCA component also indicated that the probability of a seed being removed is not independent of the main characteristics at the microhabitat scale. In all seasons, there were some differences in one or more components between the mean value of explored microhabitats and the expected mean under the null model (Fig. 4). However, evidence of selection depended on the null model assumed. In the analysis at the habitat extent (Fig. 4a), there was a preference for devices in microhabitats with less cover of shrubs and litter (PC1) in all seasons, in microhabitats with trees in spring and summer (PC2), and with less grasses (or more low shrubs) in summer (PC3). Variances of scores of used devices were no different from those expected by chance in any component or season, even when different means were detected (i.e., even when means differed, characteristics associated to used devices were as variable as those available). When the analysis controlled for the possible hierarchical selection at the grid level with a stratified null model, skewed use was still observed on PC1, favouring microhabitats without cover and litter in all season, but significant selection along PC2 practically disappeared and preference on PC3 for microhabitats with no grasses became stronger (statistically significative in spring, summer and autumn; Fig. 4b).

### Foraging site selection based on microhabitat position

**Fig. 5:**
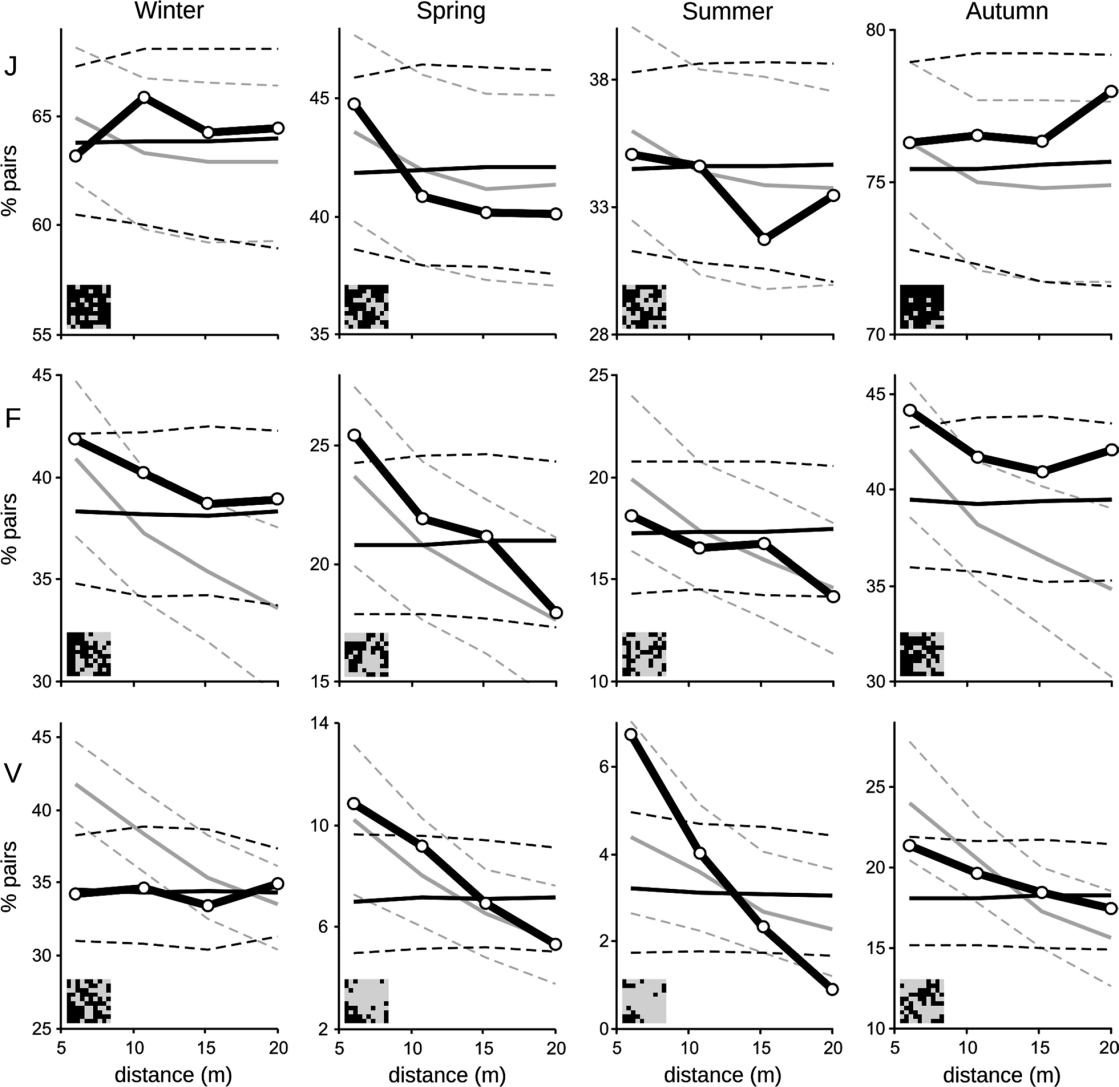
Spatial autocorrelation of seed removal. Percentage of used-used pairs of devices along four distance classes (black thick lines) in each season in three 10 × 10 grids (J, F, V). Black thin lines show the expected percentage (continuous) and 2.5% and 97.5% percentiles (broken) of used-used pairs under a Complete Spatial Randomness Model (CSR). Gray lines show the same three statistics under a spatially heterogeneous model that considers a negative influence of increasing distances to tall trees (DTT model; see details in text). Actual patterns of seed removal are shown as insets in the bottom left corner of each subfigure, with used devices in black

In several seasonal trials there was a positive spatial association of seed removal at short distances (5–7.5 m). Neighbouring devices (i.e., separated by the first distance class) were both used more frequently in spring (*P* = 0.018) and autumn (*P* = 0.023) in grid F, and in spring (*P* = 0.006) and summer (*P* = 0.001) in grid V, than expected by chance (Join-counts analyses against Complete Spatial Randomness Model, CSR; Fig. 5). Similar patterns, though statistically non-significant, were observed for other seasonal trials in the same two grids (winter in F: *P* = 0.071; autumn in V: *P* = 0.103). No significant spatial pattern was detected in grid J. Correlogram slopes (i.e., how autocorrelation changes with distance) were always steeper in spring and summer than in autumn and winter (Fig. 5).

**Fig. 6:**
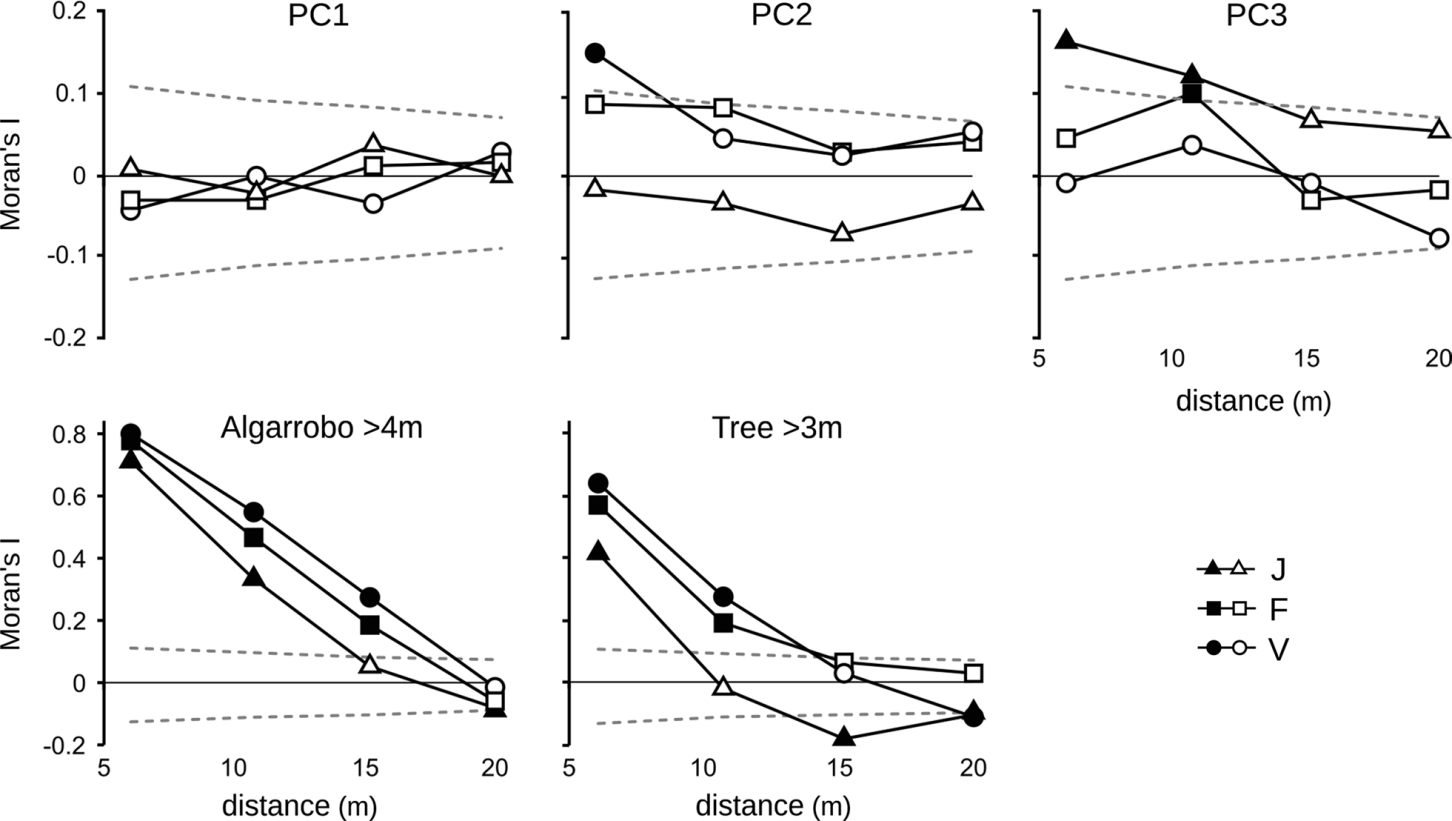
Spatial autocorrelation of microhabitat and vegetation characteristics. Correlograms of synthetic variables (after a PCA) with characteristics of the vegetation at microhabitat scale and of two variables of distance to tall trees in the three 10 × 10 grids (J, F, V). Broken lines are the 2.5% and 97.5% percentiles of the Moran’s I values expected by chance under a Complete Spatial Randomness Model (CSR); full symbols show values outside those point-wise intervals (i.e., *P* < 0.05)

Spatial aggregation of seed removal was not a direct consequence of spatial autocorrelation of vegetation characteristics at the microhabitat scale (Fig. 6): (1) PC1, the component more consistently associated with seed removal (Fig. 4) was not spatially autocorrelated; (2) PC2 was positively autocorrelated in grid V at the first distance class but was not significantly relevant in explaining seed removal once the effect at the grid scale was controlled for; and (3) PC3 was positively autocorrelated in grid J and at the second distance class in F, both at which there was no evidence of autocorrelated seed removal (Fig. 5).

In contrast, distance from devices to tall trees showed a strong positive autocorrelation (Fig. 6) as a consequence of many neighbouring devices sharing the same nearest tall tree (26.7–68.0% distances from a device to the closest tall algarrobo were > 5 m). Correlograms describing autocorrelation of seed removal were more similar to those of a heterogeneous Poisson model with tall trees as main predictor (DTT: Distance-To-Tree Model, Fig. 5). Observed correlograms in grids V and F were more similar to DTT model in spring and summer, particularly at short distances, while autumn–winter pattern were intermediate or more similar to CSR, particularly at longer distances. In grid J both models had similar predictions as a consequence of the higher abundance and more uniform distribution of tall trees; no observed pattern would have statistically distinguished between the two null models.

## Discussion

We offered scattered single seeds in seasonal trials at algarrobales in Ñacuñán (central Monte desert, Argentina) to detect the use of space by foraging postdispersal granivorous birds and analyze its association with vegetation and soil characteristics at the microhabitat scale (1–2 m diameter). Our multivariate characterization of vegetation heterogeneity proved consistent with previous studies in this habitat, including those characterizing soil seed bank abundance, composition and dynamics [21, 22, 30]. We start by discussing the experimental field technique used and the evidence it provided, then focus on our main hypotheses on selective patterns by foraging granivorous birds at the microhabitat scale and discuss selective patterns detected at bigger spatial and temporal scales, and finally conclude on the potential causes and implications of these and related results.

### Postdispersal granivory by birds as inferred from seed-offer field trials

Though several authors have assumed that desert birds rarely find, or are slow to detect, experimental seed dishes or trays (e.g., [46–48, 64]; see [50, 65]), our experimental scattered seeds were readily found and removed by local birds during the first day of every seasonal trial, even when seed reward at the patch scale and seed addition at the grid scale were nil (100 seeds in ~ 0.2 ha, or < 1.5 g/ha). The ability of small granivorous birds to frequently explore their habitats and detect scarce, dispersed seed resources should not be assumed poor in every desert, nor compulsory the alteration of their patch search patterns through training sessions or a “magic pudding effect” [31].

Control of selective access by different granivorous taxa is another typical problem in seed removal studies [65], usually involving physical barriers that may not prove neutral or successful ([66, 67], *pers. obs.*). We used a day–night criterion calibrated by recording foot tracks over smoothed soil around ant-proof seed-offer devices. In this environment, single-seed offer proved a very simple, quick, scalable and economic solution for studies that require the assumption that foragers usual use of space is not modified by the experimental setup. However, foraging patterns at the guild level may hide important variations or blur selective patterns in the use of space by some granivores when seed removal can not be assigned to particular species or individuals. Radio-frequency identification tags, radiotelemetry (e.g., [68]) or videographic techniques (e.g., [67, 69]) may be more precise or complete alternatives (see also [31]), but they are more time-consuming, expensive and limited in resolution or extent.

Seasonal variation in seed removal by birds proved consistent within and across studies, adding evidence in support of this technique to study postdispersal granivory. Seed removal was always highest in autumn and winter and decreased in spring to a minimum in summer in each grid of our field study. This agrees with direct observations of small granivorous birds foraging on the ground [30], estimations of granivory by measuring seed biomass removed from 25 to 50 ad libitum trays ([49]; figure 3.21 in [60]) and known local changes in abundance and diet of seed-eating birds (see “Granivorous birds and the soil seed bank”).

### Bird foraging and microhabitat characteristics

As a consequence of their adapted behavioural traits and their local experience, foragers should be able to track spatial variations in food abundance when coupled to perceptible environmental cues [7–9] (but see [70]). Against our bottom-up hypothesis, conceptually based on an “attack” foraging model, postdispersal granivorous birds were less selective than expected if they were avoiding patches under a profitability threshold for this environment. Variability in main microhabitat features was similar around used and available devices, suggesting that no kind of microhabitat is safe from bird exploration, at least in areas with enough resources to sustain their populations. It is not straightforward to conclude that they are unable to perceive bold characteristics of the vegetation or that their cognitive abilities do not allow them to recognize the distribution of patch qualities or develop a patch-searching rule based on them. Instead, patch value may not be a function solely of seed abundance if there are correlated factors that reduce their profitability, such as seed detectability and foraging efficiency in different substrates [59, 71–73], or involve important costs to trade-off as thermoregulation or predation risk. Milesi and Marone [23] tested for patch selection in field aviaries under varying combinations of these factors and in spite of some influence of them all, seed abundance was still the main predictor of patch exploitation by individual birds. Desert granivores must cope with a dynamic extreme system with high spatio-temporal variation while foraging for an anyway abundant and dispersed small-sized prey. This scenario may have evolutionary favoured less-informed strategies dependent on behavioural mechanisms such as learning and plasticity (e.g., sample every patch to increase survival when under extreme conditions, an opportunistic strategy) over a fixed or narrowly defined patch selection template fine-tuned to a locally set of more beneficial features (the “behavioural gambit” sensu [70]) [74–76]. The penalty of visiting many exposed areas that were effectively poor foraging patches may be counterbalanced by the occasional detection of valuable but variable ones, such as ephemeral patches of recently-fallen grass seeds or scattered depressions that accumulate seeds during secondary dispersal by wind and water (an usual feature in Ñacuñán and other deserts [18, 22]), at least when not under immediate risk of starvation [77]. Moreover, structural information embedded in vegetation may be of less value, and instant sampling required anyway [3, 13, 78, 79] if the relevant foraging scale of these birds is much smaller (see [9, 10, 80, 81]), with seed bank (within a microhabitat) then affected by accumulation gradients or micro-topography, and temporal changes from secondary dispersal and consumption.

Still, seed removal was not completely random. Birds repeated their use of experimental devices at particular positions (both in consecutive days of each trial and along the seasonal trials) in spite of intra-annual variations in seed abundance and distribution, bird abundance, guild composition and foraging behaviour, suggesting that some permanent patch features have an influence on its probability of exploration. Strikingly, the only consistent selective pattern at microhabitat scale was against our main prediction: seed removal was always skewed towards those patches with less perennial vegetation and litter cover, and in most cases with less grasses. All these characteristics associate with fewer seeds in the soil bank, supporting our hypothesis of a top-down spatial effect by birds, with seed consumption creating or strengthening (and not responding to) the spatial pattern and dynamics of the seed bank. Though not total seed abundance, several factors may favour this preferential exploration of open microhabitats. Birds may be targeting recently produced seeds that preferentially enter the seed bank through open spaces during primary dispersal in summer and autumn [57], but foraging patterns from winter to early summer do not seem to match predictions of a bottom-up model based on seed renewal rates [82, 83]. Easy accessibility between the ground and elevated perches, and open lines of sight for predator detection, may cause open microhabitats to be perceived by small birds as safer foraging spots. Woody cover may involve higher risks of predation that will trade-off against foraging related tasks, resulting in higher costs in terms of fitness [84–86]. Frequent patch visits of short duration, a scenario where profitability is less dependent on total seed abundance at microhabitat scale, may be an attempt to avoid learning predators by being less predictable in space [87, 88].

### Bigger scales: influence of context on patch exploration

Bigger spatial scales proved more relevant to describe heterogeneity in bird foraging in this habitat. Habitat selection and foraging patterns resulting from combinations of scale-dependent preference at different scales appear in most ecological situations [62, 63, 89, 90]. Seed removal differed consistently among three grids (~ 0.2 ha) that were haphazardly established in the algarrobal, with differences among grids even higher than the strikingly strong and repeatable seasonal pattern of granivory (illustrating an additional challenge for attempts at global explanations of granivory rates derived from one-shot seed-offer experiments; e.g., [91, 92]). In the most used grid (“J”) every microhabitat appeared “suitable enough” (only 4/100 devices were never used) while in the others (“F” and “V”) birds behaved more selectively: seed removal in autumn–winter showed aggregation at small distances, but in spring–summer trials birds did not visit important portions of these grids. In consequence, inferences on microhabitat selectivity on spatially autocorrelated characteristics depend on how the use of space by foraging birds at bigger scales is assumed. For example, spring–summer preference for patches under trees in the simplest analysis (at the habitat extent, i.e., pooling grids) disappeared when controlling for the differential use of each grid (either with a stratified analysis or analysing each grid separately). This influence of the scale of analysis suggests a selective use of foraging patches based not only on patch characteristics (around an experimental device) but also in its relative position among other patches or in relation to other structures.

Most notable among those influences was the negative association between seed removal by birds and distance to tall trees, both as a selective pattern among and within grids. A simple heterogeneous Poisson model in which probability of seed removal was negatively related to distance to tall trees successfully predicted the observed change in autocorrelation with distance in two grids and the lack of autocorrelation in the third. Selective foraging around tall trees was also concluded from direct field observations of these species [30], and it is not unusual elsewhere (e.g., [93]). This preferential exploration of areas close to (but not exclusively under) tall trees is probably not related to seed abundance. Though we lack hard data on heterogeneity of seed abundance at this scale (e.g., tens of metres), average seed abundance was rather similar in close habitats that differ markedly in tree cover [22, 94] and local studies found no correlation between abundances of grass seeds and granivorous birds at a slightly bigger scale (200 × 100 m [95]).

Birds may have preferred to visit shaded patches near trees, with a stronger pattern in spring and summer, for thermoregulation. Lopez de Casenave et al. [49] also found higher rates of seed removal from ad libitum trays under trees than in open patches in summer but not in winter. However, in an additional summer trial with the same experimental design but tracking seed removal in grids J and V throughout the day, we observed that most seed removal, (again skewed towards areas around trees in the more heterogeneous grid, V), occurred during the morning and afternoon and it was not related to shade (detailed results in [60]). Milesi and Marone [23] also showed that extreme temperatures may pose an ultimate limit on foraging but can not be invoked as the single main reason for spatially selective foraging patterns by individual birds.

Territoriality (reproduction) and safety (predation risk) are more probable causes of this association with tall trees. Though a typical central foraging model around nest sites cannot be invoked (most of these species nest on shrubs, on the ground or in smaller *Geoffroea decorticans* trees [96, 97]), these birds are territorial, particularly during spring and summer when tall trees are preferred singing perches [98–100]. Even when their territories in the algarrobal tend to be contiguous [101], birds may be flying to and from tall trees as important posts for antipredatory and territorial vigilance, visiting near patches more frequently. Seasonal variations in its influence on foraging may associate with changes in guild composition, movement patterns, territoriality and territory size [68, 101, 102]. Foraging far from tall trees may involve higher predation risk, as interpreted in classic studies with granivorous birds (e.g., [89, 103–106]), and also change seasonally with varying perception, evaluation or actual risk of predation [106–108]. There was some experimental [23] and observational support (as in other multi-layered habitats, most local birds flee to tall trees as refuges when disturbed; *pers. obs.*) for this predation risk hypothesis.

## Conclusions

According to their patterns of single-seed removal, the guild of granivorous birds in the algarrobal of the central Monte desert explore every kind of microhabitat. Against our bottom-up hypothesis based on a predictable and heterogeneous seed bank [21–24, 57]), birds did not rely on visual information embedded in vegetation structure and soil cover to attack only rich seed patches (behaving as “myopic foragers” sensu [1]). Conversely, seed removal was more frequent in open microhabitats, which in average contain less seeds all year round. Openness (no woody cover), probably as a cause of better accessibility at ground level or to facilitate predator detection, seems more important at this smaller scale than positive predictors of soil seed abundance. The presence of tall trees, irrespective of the main underlying cause, seems to determine a first level of selection that defines explorable space, and then microhabitat structure exerts an influence on which patches are effectively exploited (or more frequently visited), not an unusual conclusion for small ground-foraging birds (e.g., [5, 76, 109, 110]). This two-scale use of foraging space, matching the two modes of movement of these small birds within a stratified habitat (flying between perches and from perches to the ground, and walking on the ground while foraging) could result in a scenario of heterogeneous removal of seeds, particularly for the grasses these birds prefer [53, 54]. In a seed-limited environment (as this one [24]), this may cascade to a top-down effect on the spatial distribution of plant populations. Expected patterns of seed removal under this multiscale hypothesis should ideally be tested at bigger spatial extents, including higher degrees of environmental heterogeneity and more diverse distances to tall trees. Adding simultaneous estimations of bird and seed densities will allow the study of how bird foraging patterns change with granivory intensity and seed relative value as food resource to further understand the dynamics of this interaction and its consequences for desert communities.

## Methods

### Study area

The study was done in the Biosphere Reserve of Ñacuñán (34°03′S, 67°54.5′W), in the central Monte desert, Province of Mendoza, Argentina. The climate is dry, with wide variations in annual precipitation between years (mean: 348.9 mm, range: 192.6–585.4 mm, 1972–2002). It is also highly seasonal, with warm and rainy summers (> 20 °C; 269 mm) and cold and dry winters (< 10 °C; 80 mm). A complete description of the area can be found in [81]).

The main habitat of the Reserve is the algarrobal, an open woodland of algarrobo (*Prosopis flexuosa*) trees 3–6 m high scattered in a matrix of perennial *Larrea divaricata* tall shrubs (1–3 m high, horizontal cover > 35%). Other woody species are *Geoffroea decorticans* trees, tall shrubs such as *Capparis atamisquea*, *Condalia microphylla* and *Atriplex lampa* (usually > 1 m high), and low shrubs (~ 20% cover, usually < 1 m high) such as *Lycium* spp., *Verbena aspera* and *Acantholippia seriphioides*. There is also an important cover (> 25%) of perennial grasses (*Pappophorum* spp., *Trichloris crinita*, *Digitaria californica*, *Aristida* spp., *Setaria* spp., *Sporobolus cryptandrus*). Most of the reserve has been closed to cattle ranching and other significant human activities since 1971. About a third of the surface of the algarrobal lacks perennial vegetation in the form of open patches of variable size (from centimetres to metres). Forb cover is highly variable among seasons and years, usually an order of magnitude lower than grass cover. Forbs were not considered in the description of the vegetation structure, following criteria in local studies of the seed bank [21, 22] and bird foraging [30].

### Granivorous birds and the soil seed bank

The guild of small seed-eating birds that forage mainly from the ground is formed by 4–5 species: *Zonotrichia capensis*, *Saltatricula multicolor*, *Diuca diuca, Phrygilus carbonarius*, and *Poospiza ornata* only in spring–summer [30, 111, 112]. The guild changes in abundance and composition along the year: the abundant southern subspecies *Zonotrichia capensis australis* is only present during autumn and winter (other subspecies are resident) [113] and *Poospiza ornata* arrives for the breeding season [114]. We expect these species to remove experimental seeds from the ground according to their mean proportional abundances in this habitat [95]: autumn–winter: 60.4% *Z. capensis*, 23.9% *P. carbonarius*, 8.1% *D. diuca*, 7.6% *S. multicolor*; spring–summer: 49.4% *P. ornata*, 18.7% *S. multicolor*, 15.5% *D. diuca*, 11.3% *P. carbonarius*, and 5.2% *Z. capensis*. Seeds of herbaceous plants are the staple diet of these granivorous birds (75–99% of their granivorous diet is grasses and one forb species [56]). All postdispersal granivorous birds prefer medium–large grass seeds [53, 54] and reduce their seed intake in spring and summer to include insects and fruit in their breeding diets [30, 112]. Other birds able to consume seeds do not forage on the ground, are rare or only visit this habitat occasionally (e.g., *Poospiza torquata*, *Catamenia analis*, *Zenaida auriculata*, *Columba maculosa*, *Columbina picui*, *Carduelis magellanica*, *Molothrus bonariensis*, *Passer domesticus*, *Eudromia elegans*, *Nothura darwinii*; [95]).

Primary seed dispersal starts in late spring and finishes by winter, with maximum seed availability in the soil during autumn–winter and a minimum when summer begins [22, 57]. Seed abundance in the soil is very heterogeneous at small scales, with close patches of extreme abundances. Seeds are consistently more abundant under trees and shrubs and in depressions of the soil where litter accumulates, mostly because of the persistent seed bank of forb seeds [22, 24]. The abundance of grass seeds is less heterogeneous though still higher under woody cover, with some intra- and inter-annual variability. As a consequence, our hypotheses and their predictions should apply for any of the main granivorous bird species and for all of them analysed as a guild.

### Experimental offer of single seeds

Single seeds were offered on top of each of 300 devices made of upside-down feet of plastic flute glasses with their stems buried so the top (originally the base of the glass) remained 2–3 cm over the ground. This configuration prevented access by granivorous ants and other arthropods that cannot walk upside-down on the smooth plastic surface ([49, 115], *pers. obs.*). Devices were arranged 5-m apart on three 10 × 10 grids (“J”, “F” and “V”; area ≈ 2000 m^2^ each) located 80–400 m apart within the algarrobal. A single *Setaria italica* seed (a commercial species bigger than the otherwise similar local *Setaria leucopila*, both readily consumed by birds [53]) was offered on each device for two consecutive days from sunrise to sunset (standardised following data and civil twilight criteria by U.S. Naval Observatory [116]), once per season (ranging from ~ 23.3 h in Autumn to ~ 29.5 h in Summer). The top surface of the device, 6 cm in diameter, was covered with local soil for a similar visual appearance to the surrounding ground. According to estimations from simultaneous soil samples (L. Marone, unpublished), our experimental seed offer was not particularly attractive against background seed offer in this habitat: from 2.4 (bare soil in spring) to 19.3 (beneath shrubs in winter) grass seeds are expected in a similar sized area of soil, with a similar biomass density to the expected average for grass seeds during winter (~ 0.1 mg/cm^2^) and 36% of the biomass of all consumable seeds (those in the diet of granivorous birds [56]).

We were not able to identify which animal species removed the seed from each device. However, rodents that may have remove seeds in the area are mainly nocturnal [49]. Two additional trials were done under a modified protocol to test the assumption that birds were the (only) diurnal organisms removing seeds from these devices. Clayish local soil was smoothed around 50 devices in grid F before offering seeds during an extra day after the main summer and winter trials. Footprints of birds, mammals and other taxa (insects and lizards) were easily recognised, though we were not able to confidently distinguish among the focal granivorous bird species. In most cases where the seed has been removed only bird footprints were detected, in both winter (32/35 = 91%) and summer (7/9 = 78%) trials. The rest had mixed footprints of birds and other taxa or not recognisable footprints, but no device without seed had footprints of other taxa exclusively. On the other side, no bird footprints were found around devices where the seed remained, suggesting that it was not rejected when closely approached by walking birds. Moreover, removal of single seeds did not differ from removal of groups of ten seeds during a pilot study, suggesting that birds completely remove small groups of accessible experimental seeds when detected.

Since seed removal from a device was not independent between the two consecutive days in any of the four seasons (Fisher exact tests for 2 × 2 contingency tables: χ^2^ > 14.15, *P* < 0.001, *n* = 300, with more observations of “double removal” and “never removed” than expected by chance), a device was defined as “used” in each season if the seed was removed at least once during the 2 days it was offered. Independence of seed removal among seasons was tested for each grid by comparing the distribution of observed frequencies of the number of seasonal trials in which each device was used (0–4) against the expected frequencies calculated as the product of four Bernoulli trials with *n* = 100 (each seasonal experiment) with variable probabilities of success, estimated as the proportion of used devices in that grid for each season. Goodness of fit was evaluated with a χ^2^ statistic.

Temporal and spatial heterogeneity in intensity of seed removal (proportion of used devices per grid) was tested with binomial Generalised Linear Models (*logit* link) with Grid and Season as independent categorical variables. Significance of predictors was assessed comparing the change in deviance of nested models obtained through stepwise backwards elimination, asymptotically distributed as χ^2^. The ratio of deviance to degrees of freedom in the minimum adequate model was 1.74, but corrections for overdispersion did not change interpretation of results (*F*- vs. χ^2^-tests).

### Vegetation and soil characteristics at the microhabitat scale

Studies on use of space through short-term observations rely on asymmetric evidence: while patch visit can be inferred from seed removal, non-removal does not imply the patch is not to be explored eventually. This lack of a proper “no use” group should raise concerns on simple statistical analyses based on a priori classification into exclusive groups (e.g., discriminant analyses), particularly if they assume similar variability in both (homoscedasticity), or based on assigning zero probability of use (e.g., classical logistic regression). Identification of explanatory variables and predictive value of the statistical models can both suffer from the unrecognized probability of false-negatives [117–119]. Though more complex modelling strategies can incorporate or simulate incomplete evidence on absences in a spatially explicit context (e.g., species distribution and Bayesian models; see [119–121]), we opted for an indirect strategy of analysis that best matches how we developed our hypotheses and predictions. First, we detected and simplified the main structural and floristic characteristics defining habitat heterogeneity at the microhabitat scale. Then, we evaluated if those characteristics and the spatial positions of used microhabitats (i.e., those where the seed had been removed) were a random (no selection) or a skewed (selective) sample of available microhabitats. This is a similar approach to that previously reported in [30] at the smaller spatial scale of analysis.

Vegetation and soil cover around each device were measured with a vertical 1-m long pole (2 cm diameter) positioned every 10 cm along four 50-cm transects (= 20 points per microhabitat) from the device to each cardinal direction. At each point, perennial plants touching the pole were identified to genus level. The presence of vegetation > 1 m and the presence of dense litter (when it prevented from seeing the mineral soil below) or its absence (bare soil) were also recorded. Percentage cover per plant group (grasses, standing dry grasses, low shrubs, tall shrubs, and trees) and of bare soil and deep litter were calculated after those measurements.

A Principal Components Analysis (PCA) with Varimax rotation of the selected axes was done to reduce the number of dimensions of the ten variables measured at the microhabitat scale. Some variables were previously transformed (arcsin, square root or logarithm) to improve the symmetry of their distributions and then standardised into the PCA correlation matrix. Alternative analyses at the level of plant genera gave similar but noisier results on the main axes. Three components (PC1–PC3) were retained following the Kaiser criterion (eigenvalue > 1), the broken-stick model, and the scree-plot [122]. Before multidimensional analyses, scores on each axis were multiplied by its eigenvalue to weight them according to the variability they represent (see [123]); though applied for correctness, these variable transformations and weights did not change results significantly. Separate PCA analyses for each grid resulted in similar principal components and scores correlated with those of the grouped analysis (Pearson correlation, all cases *n* = 100, *r* > 0.8, *P* < 0.001, except for PC3 in grid V: *r* = 0.21, *P* = 0.03) confirming that heterogeneity of main characteristics at this scale were similar for the three sites. Therefore, only PCA scores based on all microhabitats were used for subsequent analyses. Scores based on microhabitat characteristics were compared among grids with Kruskal–Wallis tests.

Microhabitats around seed devices were also categorized at field a priori following the same criteria used on previous studies [21, 22]: beneath trees, beneath tall shrubs, beneath low shrubs, beneath grasses (under no woody cover), and bare soil (no perennial cover). Microhabitats around 79 grid points (26%) did not fit neatly into any of those categories and were assigned to an “intermediate” category (e.g., shrub borders).

Big trees (trees > 3 m and algarrobos > 4 m high) in and around the grids were mapped, measuring the distance between each device and the nearest tree canopy. Other minimum distances to vegetation in several height strata and to closest canopy of each plant group were measured but resulted highly correlated with measurements of horizontal cover (since most distances were smaller than a microhabitat radius, e.g., 96% of distances to nearest grass or 78% to vegetation 1–2 m high, see [60]). Therefore, only distances to tall trees were informative in addition to the measured characteristics at the microhabitat scale. Distances were compared among grids with Kruskal–Wallis tests.

### Foraging site selection based on microhabitat characteristics

Selection at the microhabitat scale was evaluated (1) multidimensionally, with a spatial technique applied to results of the PCA ordination and (2) unidimensionally, with a randomization test for each of the three retained PCA components. The first test is analogue to the representation of used and available microhabitats in multidimensional scatterplots and the evaluation of the spatial segregation between two classes of points. The test was a 3-D extension of a 2-D point pattern spatial analysis that classifies each point by its type and that of its nearest neighbour and compares the proportion of each kind of pair with that expected by chance (i.e., a join-counts analysis of a binary label according to a nearest-neighbour matrix, testing for differences against a random labelling model [124, 125]). A number of pairs of equally labelled points greater than expected by chance indicates that used or available points were aggregated in the PCA space. Global spatial segregation between classes of points was evaluated with a 2 × 2 contingency table, with expected frequencies and statistic (*C*, asymptotically distributed as Chi-square with 2 d.f.) as proposed by Dixon (NN test [126]). When significant evidence of global segregation was found, each type of pair was tested with an asymptotically normal *Z* statistic [126]. Statistical analysis were done in R [127], modifying the functions provided by Dixon [128] to a multidimensional case to obtain the matrix of Euclidean distances between points and identify nearest neighbours in a 3-D case.

To evaluate selection on each principal component, the null hypothesis that microhabitat characteristics of used devices are a random sample of those of the available ones was tested with randomization tests [129–131]. Statistics of central tendency (mean, median) and dispersion (variance) were calculated from 4999 or 1999 samples, respectively, of the same size as observed (used), taken without replacement from the available values, to evaluate selection consisting in a skewed use of lower or higher values of the environmental variable (resulting in lower or higher mean or median) and selection consisting in avoiding extreme or central values (lower or higher dispersion, respectively; see [120, 132, 133]). Results based on the median of the distributions (not reported) were very similar to those based on the mean. A pseudo-*P* value associated to the hypothesis that the observed statistic was obtained by chance was calculated as double the number of equal or more extreme values than the observed in the distribution, divided by the number of samples taken including the observed (i.e., a two-tailed test). PCA scores were spatially independent on most axes (see “Results”), so no correction was applied to statistically evaluate selection hypotheses at the microhabitat scale.

Two sets of confidence intervals and probabilities were calculated, based on different null models. First, randomly chosen values were obtained in each iteration from the 300 values of all available microhabitats to evaluate selection assuming no selective use of space at bigger scales (up to the extent of the study). Second, a stratified null model was done to control for a possible habitat selection at the grid scale (i.e., assuming a hierarchical use of space based on the observation that grids differed systematically in the proportion of used devices, see “Results”). Under this model, random samples of the same size as observed were taken from each grid, so the expected mean value was an average of the mean values of the three grids weighted for the number of used devices in each of them.

### Foraging site selection based on device position

Seed removal in each 10 × 10 grid was examined for spatial autocorrelation using spatial analyses for non-continuous data, assuming an isotropic process. Spatial autocorrelation of seasonal seed removal and of microhabitat characteristics were evaluated comparing a measure of similarity between pairs of points given by their position with another determined by the focal variable (agreement between two matrices of similarity [124]). Discrete Euclidean distances between devices (from regular grids) were aggregated in four distance classes: < 8.5 m (nearest 8 neighbours of a focal central grid point), 8.5–12.5 m (12 neighbours), 12.5–17 m (16), and 17–22 m (24). Relationship between points given by distance (known as matrix of weights, *W*) were considered binary, with 1 indicating that two points were separated by a distance in the focal distance class, and 0 otherwise. Two similar spatial analyses were used according to the type of variable [124, 134, 135]: join-counts for binary variables (seasonal seed removal in each distance class) [136], and Moran’s *I* statistic for continuous variables (values of the main PCA axes, distance to trees). Correlograms from both analyses can be interpreted in a similar way: values higher than expected indicate positive spatial autocorrelation at the focal distance class (i.e., aggregated pattern), and values significantly low indicate negative spatial autocorrelation (i.e., overdispersed or regular pattern).

The number of pairs of devices that were both used (1–1 joins) was compared against expectations from two different models: (1) Complete Spatial Randomness (CSR), in which the probability of use of a device follows a homogeneous Poisson process depending only on the observed number of devices used in a grid, with no spatial interaction (i.e., all devices have the same chance of being used independently of its neighbours), and (2) Distance-To-Tree Model (DTT), a very simple heterogeneous Poisson producing aggregation of seed removal from first order or induced autocorrelation associated with distance to tall trees. This analysis tests the observed configuration keeping the observed composition, edge effects and potential habitat selection at bigger scales (i.e. different use of grids), assuming no second order autocorrelation (i.e., the use of a device is independent of that of its neighbours except for the modelled first order autocorrelation). The expected distribution of the statistic under each model was estimated with 1999 random samples of grid points of the same size as observed (with no replacement). For CSR, all points shared the same probability to be selected. For DTT, probability of use varied inversely with distance to the nearest tall tree: a device had a relative probability of use of 0.6 if at < 5 m or 0.3 if at 5–10 m of an algarrobo > 4 m high and a probability of 0.1 if at < 10 m to the nearest tree > 3 m high; points at > 10 m of any tall tree (none in grid J, 4 in F and 26 in V) had no chance of being selected (probability = 0). Expected values (median) and limits of confidence intervals (percentiles 2.5% and 97.5%) were obtained from each of the generated distributions, estimating the probability *P* that the observed value belongs to the distribution under the expected model as two times the proportion of equal or more extreme values than the one observed, including it (i.e., a two-tailed test with *n* = 2000). Simple algorithms for resampling and join-counts estimation were programmed in R [127]. Moran’s *I* statistics for continuous variables were analysed in a similar way, comparing them against a CSR as implemented in the software passage [136]. Complementary spatial analyses with alternate techniques (SADIE [137]) provided similar evidence (see Additional file 1, and [60] for details).

## Additional file

**Additional file 1.** Red–blue plots mapping the spatial pattern and spatial association of seed removal according to SADIE (Spatial Analysis by Distance IndicEs [137]) for spatially referenced count data. (a) Spatial pattern of seed removal for each seasonal trial, and spatial association between consecutive trials. Each circle represents one of the 300 seed-offer devices arranged in three 10 × 10 grids (F, J, V). Red full circles belong to clusters or patches of seed removal (local indices of aggregation higher than 95% of positive indices obtained after 5850 permutations); blue full circles belong to gap clusters (index < 95% of negative values). Empty circles with thick border represent indices within the 90–95% ranges (marginal), and empty thin-bordered circles represent non-significant aggregation indices (closer to 0 than the 90th percentiles). Significative global spatial pattern is shown over each grid, showing its direction (+: more clustered than expected by chance, −: overdispersed) and pseudo P-value. Arrows represent significative spatial (+: positive, −: negative) association between spatial patterns of seed removal in consecutive trials (although the rightmost arrow shows spatial association between the non-consecutive autumn and winter trials), with pseudo *P*-values (two-tailed test). Absence of values and arrows indicate degrees of clustering and associations not different from those expected by chance (*P* > 0.1). (b) Spatial pattern of seed removal (patches and gaps) for all trials combined (a count variable from 0 = seed was never removed to 4 = seed was removed in all seasonal trials). Same references as above. (c) Actual seed removal for all seasons combined and distance to tall trees. Circle size is proportional to the number of trials in which the seed was removed, with full quarters indicating the season(s) in which the device was used (clockwise from winter on top). Dark and mid green represent areas closest than 5 m and between 5 and 10 m, respectively, to the nearest tall (< 4 m) *Prosopis* tree, and light green areas < 5 m from any > 3-m tall tree.

## Data Availability

The dataset generated during the current study is available in the Zenodo repository at 10.5281/zenodo.1219825.

## Abbreviations

CSR: Complete Spatial Randomness Model
DTT: Distance-To-Tree Model
GLM: Generalised Linear Model
PCA: Principal Components Analysis
