# Supplementary figures and images for: Are all patches worth exploring? Foraging desert birds do not rely on environmental indicators of seed abundance at small scales
Source: BMC Ecol. 2019 Jun 18;19:25. doi: 10.1186/s12898-019-0242-z (PMC6582492; doi:10.1186/s12898-019-0242-z)

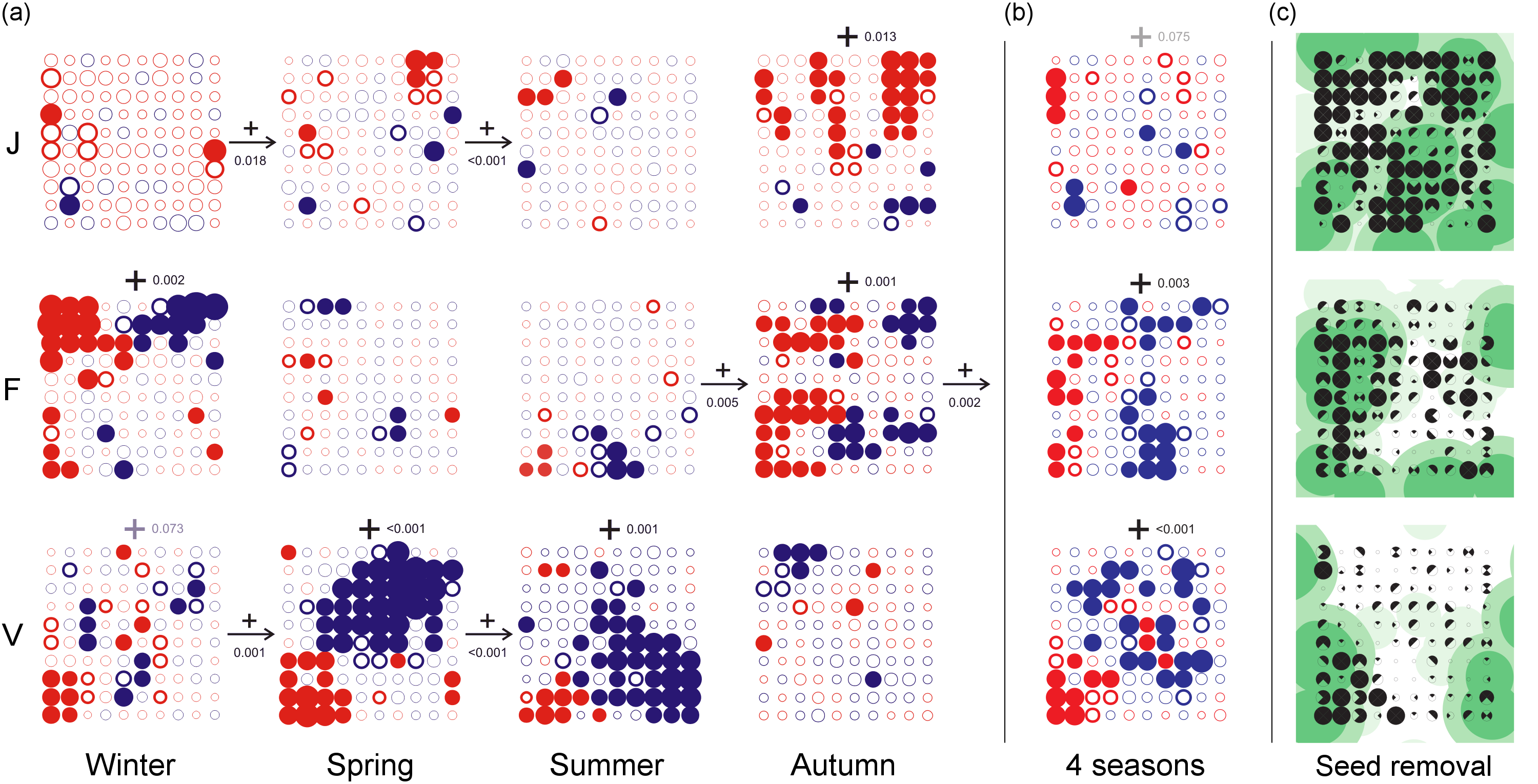

Supplement: Supplementary file 1 — Additional file 1. Red–blue plots mapping the spatial pattern and spatial association of seed removal according to SADIE (Spatial Analysis by Distance IndicEs [137]) for spatially referenced count data. (a) Spatial pattern of seed removal for each seasonal trial, and spatial association between consecutive trials. Each circle represents one of the 300 seed-offer devices arranged in three 10 × 10 grids (F, J, V). Red full circles belong to clusters or patches of seed removal (local indices of aggregation higher than 95% of positive indices obtained after 5850 permutations); blue full circles belong to gap clusters (index < 95% of negative values). Empty circles with thick border represent indices within the 90–95% ranges (marginal), and empty thin-bordered circles represent non-significant aggregation indices (closer to 0 than the 90th percentiles). Significative global spatial pattern is shown over each grid, showing its direction (+: more clustered than expected by chance, −: overdispersed) and pseudo P-value. Arrows represent significative spatial (+: positive, −: negative) association between spatial patterns of seed removal in consecutive trials (although the rightmost arrow shows spatial association between the non-consecutive autumn and winter trials), with pseudo P-values (two-tailed test). Absence of values and arrows indicate degrees of clustering and associations not different from those expected by chance (P > 0.1). (b) Spatial pattern of seed removal (patches and gaps) for all trials combined (a count variable from 0 = seed was never removed to 4 = seed was removed in all seasonal trials). Same references as above. (c) Actual seed removal for all seasons combined and distance to tall trees. Circle size is proportional to the number of trials in which the seed was removed, with full quarters indicating the season(s) in which the device was used (clockwise from winter on top). Dark and mid green represent areas closest than 5 m and between 5 and 10 m, resp [file 12898_2019_242_MOESM1_ESM.tif]
